# Detecting Vibrational Energy Transfer into an Enzyme Active Site via a Transition State Analog

**DOI:** 10.1021/acs.jpclett.6c01422

**Published:** 2026-06-26

**Authors:** Jan Gerrit Löffler, Erhan Deniz, Yunxia Shen, Carolin Feid, Jens Bredenbeck

**Affiliations:** Institute of Biophysics, Johann Wolfgang Goethe-University, Max-von-Laue-Straße 1, 60438 Frankfurt am Main, Germany

## Abstract

Vibrational energy transfer (VET) in enzymes has been discussed in the context of enzyme catalysis. A key point for experimentally investigating VET is to position suitable vibrational energy sensors in the delicate active sites of enzymes without disturbing their native state. Ideally, native substrates and/or inhibitors can be repurposed as vibrational energy sensors. Here, we demonstrate that the azide anion (N_3_^–^), bound as an inhibitor to the active site of formate dehydrogenase (FDH), sensitively detects the vibrational energy, even though it is not covalently bound. Vibrational energy has been site-specifically injected into the enzyme via exciting an azulenylalanine (AzAla) energy donor, which has been introduced by genetic code expansion in a 19 Å distance. This proof-of-principle experiment opens the field toward the investigation of VET on a picosecond time scale in a range of enzymes by exploiting their natural affinity toward the infrared-absorbing small ligand azide.

## Graphical abstract

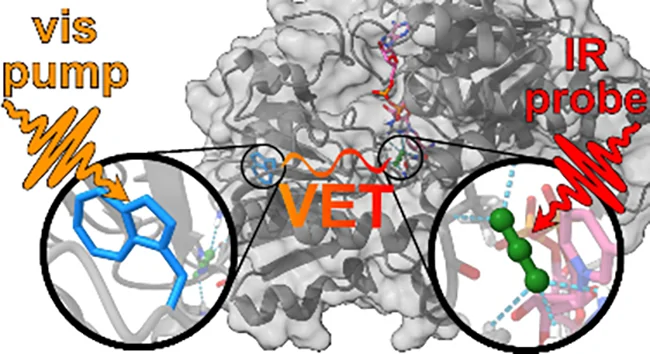

The role of protein dynamics in enzymatic catalysis remains a subject of continued debate.^1–6^ In both theoretical and experimental work it has been suggested that enzyme motions across a wide range of time scales contribute to catalytic efficiency.^1,7^ This range extends down to femtosecond-to-picosecond protein motions, which have been proposed to play an active role by facilitating barrier crossing during enzymatic reactions.^1,8–13^

A hypothesis emerging from this dynamic view is that enzymes have evolved pathways for efficient anisotropic energy transfer between solvent and active site.^1,14–16^ The redistribution of energy is mediated by low-frequency vibrational modes (LFMs), collective motions involving many residues,^17^ allowing thermal energy to be directed toward the catalytic center where it may influence barrier crossing. Simulations suggest that certain low frequency modes may directly couple to the enzymatic reaction coordinate at the active site and thereby promote catalysis.^10,12,13,15,18^ Such motions have been termed rate-promoting vibrations.^10,12,15,18^ LFMs of proteins in aqueous solution were believed to be overdamped, but an increasing number of studies indicate the occurrence of underdamped LFMs under equilibrium conditions.^9,19–21^ Yet, if LFMs are indeed directly involved in enzyme catalysis, remains controversial.^5^

While theoretical frameworks describing these effects have become increasingly sophisticated, experimental validation—particularly the direct observation of the anisotropic flow of vibrational energy into and out of the active site of an enzyme—remains a formidable challenge because of the high temporal and spatial resolution required.

Here, we report an experimental approach to probe vibrational energy transfer pathways into enzyme active sites. We show that the azide anion (N_3_^–^), a known inhibitor or transition-state analog for a range of enzymes,^21,24–33^ can serve as a minimally invasive vibrational reporter for vibrational energy transfer even though it is not covalently bound at the active site. The azide asymmetric stretch absorbs in a transparent region of the protein infrared spectrum.^24,34,35^ Using this vibrational reporter, energy arriving at the catalytic center can be monitored directly, enabling the mapping of anisotropic VET pathways within the protein. As a model system, we demonstrate the applicability of this approach in formate dehydrogenase (FDH), establishing a strategy to measure how vibrational energy propagates through an enzyme and reaches its active site.

FDH catalyzes the hydride transfer from formate to NAD^+^ and has been used for regenerating NAD­(P)H cofactors in biotechnological applications.^36–38^ FDH from *Candida boidinii* was the first enzyme, in which LFMs were observed with two-dimensional infrared spectroscopy.^20^ Furthermore, the kinetic isotope effect (KIE) of the catalytic reaction becomes temperature-dependent upon isotopic labeling of the protein^39^ or by inserting a mutation that increases the space available for the reactants within the enzyme’s active site.^34^ A temperature-dependent KIE is widely interpreted as indication for the coupling of LFMs to the catalyzed reaction.^9–11,40,41^ While the azide anion N_3_^–^ itself is not a substrate for FDH, it is known as tight-binding inhibitor and transition state analog in the noncovalent ternary complex with FDH and NAD^+^ (Figure 1).^20,42^ In this complex, the asymmetric stretching vibration of N_3_^–^ is modulated by periodic motions of the protein with frequencies of 10 and 24 cm^–1^ as measured by Cheatum and co-workers by 2D-IR spectroscopy.^20^ Evidently, N_3_^–^ senses the protein’s LFMs.

**Figure 1:**
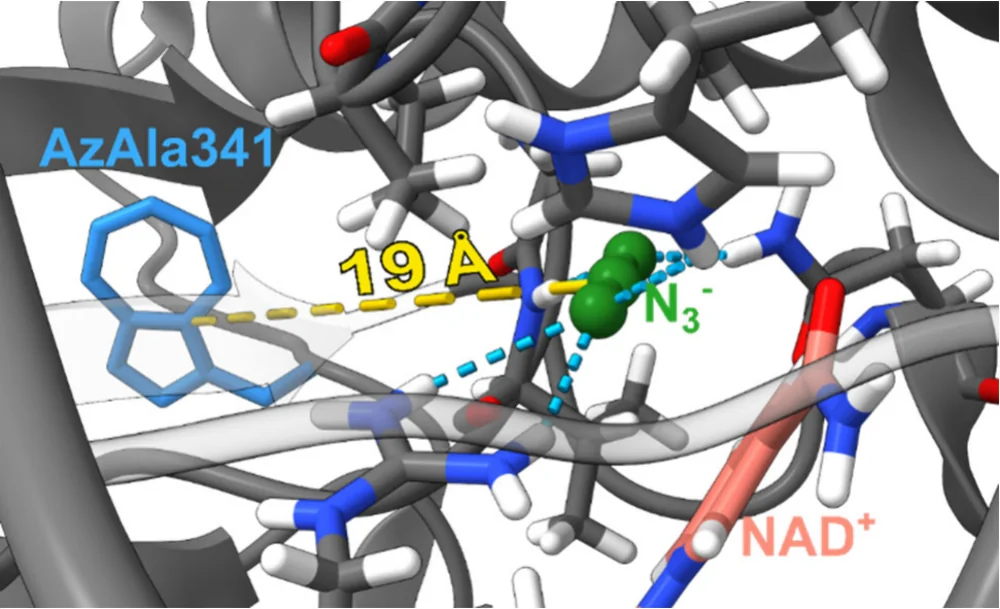
Active site view of the ternary FDH/NAD^+^/N_3_^–^ complex. The protein backbone is shown as a gray cartoon, while residues involved in N_3_^–^ binding are shown as sticks. AzAla341 is shown in light blue sticks and N_3_^–^ in a green ball-and-stick representation. The carbon atoms of the NAD^+^ cofactor are colored in salmon. The linear distance between AzAla341 and N_3_^–^ (19 Å) is indicated with a dashed yellow line. H-bonds involved in N_3_^–^ binding are indicated with dashed blue lines. Some parts of the protein in the front are semitransparent for clarity. The image is based on PDB: 5DN9 ^22^ and was created in ChimeraX.^23^

This known coupling renders the N_3_^–^ in FDH the perfect showcase for a proof-of-principle experiment in which a low-molecular substrate analog of an enzyme acts as a vibrational energy sensor. In contrast to the use of noncanonical amino acids (ncAAs) as VET sensors,^43–47^ this allows to place the sensor directly in an enzyme’s active site without perturbation by mutation.

As a vibrational energy donor, we have equipped FDH with β-(1-azulenyl)-alanine (AzAla)^48,49^ via stop-codon suppression.^44^ This tryptophan analog can site-specifically inject vibrational energy into the protein by converting the photon energy of the S_1_ excitation into vibrational energy via internal conversion on a subpicosecond time scale.^43,50^ Afterward, the energy gets redistributed into LFMs. Their couplings allow for the energy to spread further through the protein. At some point a part of that energy leads to population of LFMs, that are anharmonically coupled to the sensor mode,^51,52^ in the present system that is supposed to be the N_3_^–^ asymmetric stretching vibration.^51,52^ The coupling leads to a spectral shift of the absorption which returns to its original wavenumber when the local population of LFMs decays.

FDH is a homodimer.^53,54^ After the expression of the mutant, there are two AzAla and two N_3_^–^ present in each dimer. We chose tyrosine 341 as mutation site in our experiments, because it is on the opposite side of the protein relative to the dimerization interface (Figure S1). The through-space distance to the nearby active site is 19 Å (Figure 1) and 54 Å for intersubunit VET. At such a large separation, any intersubunit VET signal is expected to be substantially weaker and significantly delayed relative to the response of the nearest AzAla-azide pair. Consistent with this expectation, no additional delayed component is detected in the experimental VET trace. The conservative Y341AzAla mutation minimizes the possible contribution of intersubunit VET. A systematic study to map the VET pathways will require measurements of multiple energy donor sites, including those leading to VET to both active sites.

The correct function and folding of the FDH mutant were verified by an enzyme activity assay and circular dichroism (CD) spectroscopy, respectively (see Figure S2). Binding of N_3_^–^ is evident from the FTIR band shape. The spectrum of free N_3_^–^ in aqueous buffer can be described with a single Lorentzian function (central wavenumber (ω_c_): 2048 cm^–1^, fwhm: 27 cm^–1^; Table S1 in the Supporting Information; Figure S3a). FDH-Y341AzAla was prepared with an excess of NAD^+^, before adding N_3_^–^ in two steps to ensure complete saturation of the enzyme with N_3_^–^ (Figure S3b). When the N_3_^–^ is bound to FDH, the main absorption becomes narrower, and a weak side feature (16% of bound N_3_^–^) emerges (Figure 2; Table S1 in the Supporting Information). This may indicate a small fraction of N_3_^–^ that binds in a different geometry.

**Figure 2:**
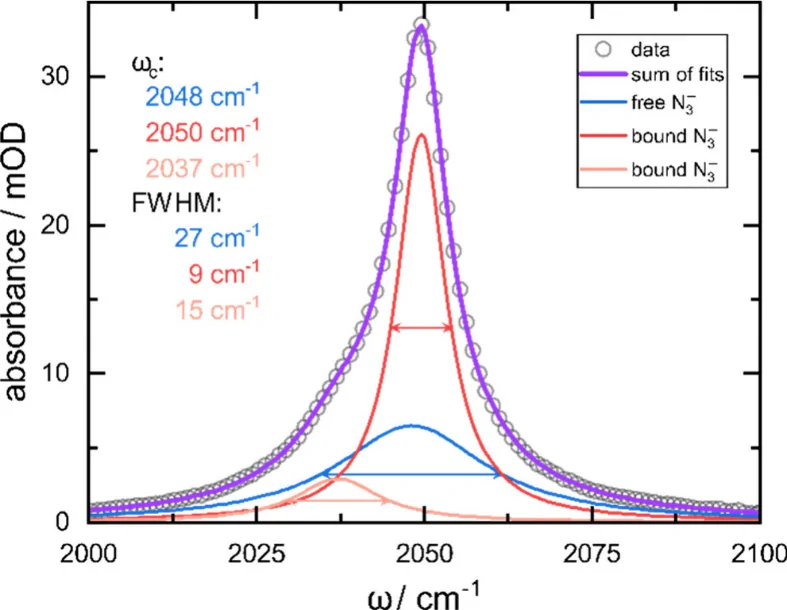
IR absorption of N_3_^–^ bound to FDH-Y341AzAla/NAD^+^. The peak can only be fully described with the sum of three Lorentzians to account for all spectral features of free and bound N_3_^–^.

In an ultrafast transient VIS-pump/IR-probe (time resolved infrared, TRIR) experiment, we excited the AzAla in FDH-Y341AzAla with 613 nm light and probed the absorption of the asymmetric stretching vibration of bound N_3_^–^ at different delays (Figure 3). Ultimately, the energy dissipates to the solvent, inducing an additional broad background signal from the underlying combination band of water. To correct for this background signal, a quasi-simultaneous measurement of the protein without N_3_^–^ was conducted in a split-sample-cell approach^46^ and subtracted from the compartment with N_3_^–^ (Figure S4).

**Figure 3:**
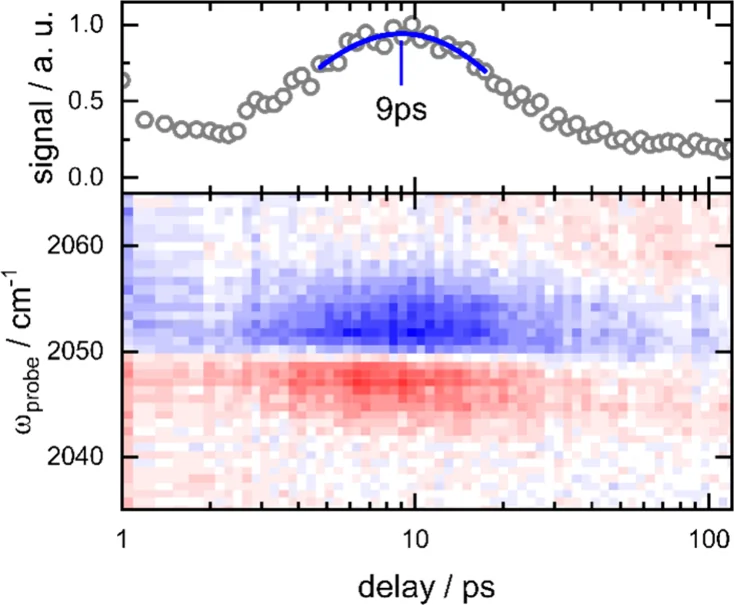
VET signal of N_3_^–^ bound to FDH-Y341AzAla/NAD^+^. The lower panel shows the N_3_^–^ difference signal induced by exciting the AzAla. The color scale ranges from −20 to 20 μOD. The redshift of the band is evident from the spectrally separated increase (red) and decrease (blue) of the absorption. The upper panel shows the integrated signal at each delay (gray circles) and a fit to determine the indicated time of the maximum signal.

In the TRIR spectra, i.e., pump-on minus pump-off, we observe a transient redshift of the N_3_^–^ band as a positive and negative feature (Figure 3). This signal reaches its maximum at 9 ps with an amplitude of 40 μOD. Notably, this peak time observed at a distance of 19 Å extends the range of our previously reported VET peak times of 3.5–7.5 ps observed at shorter distances in tryptophan zippers^45^ and PDZ3.^44,47^ The corresponding effective propagation speed of roughly 2 Å ps^–1^ is consistent with the previously reported correlations between transfer distance and VET peak time in proteins observed by us as well as by Mizutani and co-workers who used a related spectroscopic approach based on heme excitation and resonance Raman probing of tryptophan residues.^55^ The fastest peak time we observed so far (2.4 ps) was found for the AzAla donor being linked directly to a second amino acid carrying an azide sensor in a dipeptide. For this short distance, the contribution of the actual transport time is minimized and the finite time of internal conversion and energy relaxation into the low frequency modes responsible for the transport become noticeable. The increase in peak time observed for larger separations between VET donor and sensor can be attributed to the actual transport process.

The temporal and spectral features of the observed VET signal show that it indeed arises from the bound N_3_^–^ in FDH rather than free N_3_^–^ in the bulk solvent. Free N_3_^–^ would yield a VET signal with the same dynamics as the water heating signal, as it reports the energy dissipation from the protein into the bulk solution. On the time scale of our experiment, the water signal rises and then remains constant at long delays (see Figure S4b). The observed VET signal, however, shows clear rise-and-decay dynamics, showing that it indeed arises from bound N_3_^–^ inside the protein. Furthermore, the separation Δ*x* ^′^ between the maximum and the minimum of a difference signal from a shifting Lorentzian (the origin of our VET signal) with fwhm *w* cannot be smaller than (see the Supporting Information)­

$$\Delta x_{\min}^{\prime }=\frac{w}{\sqrt{3}}\approx 0.58w$$ 1

which is the limit for a very small shift causing the difference signal. Consequently, for a VET signal originating from free N_3_^–^, Δ*x* ^′^ would be at least 15.6 cm^–1^. Instead, we observe a peak separation of only 5.4 cm^–1^ (Figure S5), in good agreement with the theoretical minimum of 5.2 cm^–1^ for bound N_3_^–^. The VET signal peaks when the LFMs that couple to the bound N_3_^–^ are maximally excited.

Note that VET experiments to date have used vibrations of covalently bound probes such as azide (−N_3_) and nitrile (−CN).^47^ It is worth mentioning that the strong response of the noncovalently bound N_3_^–^ to VET is remarkable and far from obvious. N_3_^–^ has only four vibrational normal modes due to the small number of atoms.^56^ The degenerate bending mode (639 cm^–1^), the symmetric stretching mode (1364 cm^–1^) and the asymmetric stretching mode (2050 cm^–1^),^57^ are too high in energy to be directly excited by VET over a distance of 19 Å, thus anharmonic coupling from protein modes to the asymmetric stretching vibration have to be responsible for the VET signal.

Compared to our previous work with the ncAA azidohomoalanine (Aha) as VET sensor,^44^ the signal of the bound N_3_^–^ in FDH is stronger by a factor of ∼4 after covering twice the distance (and correcting for concentration and pump pulse energy). Differences in the coupling constants of Aha and bound N_3_^–^ to LFMs are not readily accessible. However, if the induced shift is similar for both sensors, the narrower bandwidth of the asymmetric stretch in N_3_^–^ would increase the signal compared to Aha. In addition to that, the extinction coefficient of N_3_^–^ is 3–4 times larger than that of Aha.^33,35,58^ Together with the fact, that N_3_^–^ does not show pronounced Fermi-resonances, a common occurrence in organic azido-compounds such as Aha,^59–62^ the N_3_^–^ anion is a preferable VET sensor.

Previous VET studies utilized native protein cofactors, e.g., heme^55,63^ or FMN,^64^ as vibrational energy donors to investigate energy dissipation out of active/binding sites. With the approach presented here, the reverse process becomes available, the investigation of energy transfer toward ligand binding/active sites of proteins. For mapping the according VET pathways, the energy donor must be placed at different sites of the protein while preserving its structure and function, which can be checked by a combination of structure-sensitive methods and functional assays. In contrast to monomeric proteins, dimeric proteins, such as FDH, require a larger set of VET measurements with different donor locations and careful data analysis to establish a clear VET map, particularly in those regions of the protein where VET to both active sites might occur. Now, with N_3_^–^ being established as a promising vibrational energy sensor, a large number of protein families, for instance heme-proteins^35,65^ and various metalloenzymes^21,28,66^ become amenable for VET studies, as they bind N_3_^–^ at their metal centers, where it routinely serves as an infrared spectroscopic probe.^21,28,35,65,66^ Whether vibrational energy transfer to the active-site ligand proceeds via its coordinating bond to the metal or other protein–ligand interactions will be the subject of future studies. The list of systems accessible for the present approach to investigate VET becomes even longer when IR-labeled substrate surrogates are considered.^67,68^

In summary, we observed VET from a genetically encoded AzAla as a vibrational energy donor into FDH’s unaltered active site, where N_3_^–^ serves as a noncovalently bound vibrational energy sensor. This ligand mimics the transition state of the catalyzed reaction, minimizing the deviation from the biologically relevant setting. For upcoming VET studies, N_3_^–^ combines the perks of covalent azido probes (large extinction coefficient) with that of covalent nitrile probes (narrow band shape),^47,69^ without the strong Fermi-resonances often observed in organic azides (for a quantitative comparison, see Table S2 in the Supporting Information). While the bound N_3_^–^ is an exceptionally simple and strong-absorbing ligand, our proof-of-principle study encourages the investigation and usage of other small, noncovalently bound vibrational energy sensors, be they native, modified, or artificial, for mapping energy transfer pathways into the active sites of enzymes.

## Experimental Methods

### Protein Expression

For the expression of the proteins, the gene of *C. boidinii* FDH (European Nucleotide Archive accession number: KM454879) was synthesized by the Invitrogen GeneArt services (Thermo Fisher Scientific). It was transferred into the multiple cloning site of an empty pET28b vector between the XhoI and NcoI cleavage sites using standard subcloning techniques and verified via Sanger sequencing.

The C-terminal hexahistidin-tag (His-tag) of the plasmid was connected to the protein by site-directed mutagenesis of an ochre stop-codon to TCA, resulting in a linker of the amino acid sequence SLE between protein and tag. For the incorporation of AzAla, the TAC codon of Tyr341 was mutated to the amber stop codon TAG. All mutagenesis were completed with the Q5 Site-Directed Mutagenesis Kit (NEB) and verified via Sanger sequencing.

The plasmid of wild-type FDH was transformed into *E. coli* BL21 Star (DE3) (Invitrogen). *E. coli* B-95.ΔA^70^ (RIKEN BRC, catalog number: RDB13711) with a plasmid containing the orthogonal pair for the incorporation of AzAla at the amber stop codon^44^ were transformed with the plasmid carrying the gene for FDH-Y341AzAla.

Expression cultures in terrific broth (TB) medium were incubated at 37 °C until they reached an OD_600_ of 0.6–1 OD. The expression was induced by addition of 1 mM IPTG, and the expression continued overnight. All media contained 50 μg/mL kanamycin to select for the pET plasmid. The expression medium for the expression of FDH-Y341AzAla additionally contained 100 μg/mL ampicillin and 1 mM AzAla. The AzAla has been enzymatically synthesized in our laboratories using previously described procedures.^71^

The cells were harvested at 8000 rpm and 4 °C, resuspended (50 mM potassium phosphate buffer [KP_i_] pH 8 with 100 mM KCl and 20 mM imidazole) and lysed via ultrasonication. DNA was precipitated using 0.5% w/v streptomycin sulfate. Cell debris and DNA were removed via ultracentrifugation at 35000 rpm for 45 min at 4 °C. The supernatant was filtered and applied to a Ni-NTA column. Only FDH-Y341AzAla was unfolded with 7 M urea before the chromatography, which allowed the separation of residual truncation product (without His-tag) that formed dimers with the full-length protein that bound to the column. The full-length protein was refolded on the column by removing the urea prior to elution with 150 mM imidazole. The unfolding/refolding steps were skipped for the wild-type protein, for which no truncation occurred. The protein-containing fractions were pooled and dialyzed against 100 mM KP_i_ pH 7.5. The protein was concentrated in a centrifugal concentrator to ∼200 μM and frozen at −70 °C until further usage.

### Sample Preparation for Laser Measurements

The protein FDH-Y341AzAla was thawed on ice and concentrated in a centrifugal concentrator at 4 °C to ∼1.66 mM. 8 mM NAD^+^ were added from a 100 mM stock solution in KP_i_. At this stage, the sample was divided into two equal fractions. To one fraction a total of 1.8 mM NaN_3_ was added from a 100 mM stock solution in KP_i_; the same volume of KP_i_ was added to the other fraction, resulting in a final protein concentration of ∼1.5 mM for both samples. Both fractions were loaded into a split-sample cell with a 100 μm spacer. The sample cell and the experimental setup has been thoroughly described elsewhere.^46^

### Laser Spectroscopy and Data Analysis

For the laser measurements, 20 μJ pump pulse energy and a spectrometer grating with 300 l/mm were used. For each compartment the signal at −40 ps delay was subtracted to remove long-lived signal contributions. Subsequently, the signal of the compartment without N_3_^–^ was fit to the signal of the compartment with N_3_^–^ at each delay with a scaling and an offset parameter to correct for the water background. Pixels carrying the VET signal of N_3_^–^ (2041–2061 cm^–1^) were excluded from the fit.

For each delay, the absolute values of the pixels carrying the VET signal were integrated to yield the time trace (Figure 3 top panel), which was then normalized to the maximum. The peak time of the normalized trace was determined by fitting a biexponential function to the data around the peak with a relative amplitude >0.65.

The separation of the extrema (i.e., Δ*x* ^′^) was quantified by fitting the sum of two Lorentzian functions with areas 1 and −1 and fwhm 8.87 cm^–1^ to the signal at 9 ps to interpolate wavenumbers between the pixels.

### FTIR Spectroscopy

The extinction coefficient and integrated extinction coefficient of N_3_^–^ (1600 M^–1^ cm^–1^ and 65.5 mM^–1^ cm^–2^) were determined from a concentration series of NaN_3_ in KP_i_ and used to determine N_3_^–^ concentrations, assuming the same integrated extinction coefficient for the free and bound species. The presented FTIR spectra of the FDH/NAD^+^ complex with and without N_3_^–^ were recorded from the samples used for the pump probe measurements on a Tensor 27 FTIR spectrometer (Bruker) with 1 cm^–1^ resolution in a 60 μm groove cuvette. The underlying water band was removed by scaling and subtracting the spectrum of FDH/NAD^+^ together with a linear baseline.

### CD Spectroscopy

CD spectra were recorded on a J-720 spectrometer (Jasco) in a 60 μm groove cuvette, shifted to 0 at 260 nm and normalized to the signal at 220 nm.

## Supplementary Material



## Notes

Derivation of Δ*x*_*min*_^′^; the structure of the FDH dimer in complex with NAD^+^ and N_3_^–^; CD spectra of wild-type FDH and FDH-Y341AzAla; FTIR spectra and fits of free N_3_^–^ and the protein samples; the background correction of the TRIR data; a plot of the TRIR signal at 9 ps with a fit from which the separation of the extrema was determined; fitted parameters of the FTIR absorption bands; and comparison of the spectral properties of N_3_^–^ and ncAA VET sensors (PDF)
