# Supplementary material for: Detecting Vibrational Energy Transfer into an Enzyme Active Site via a Transition State Analog
Source: J Phys Chem Lett. 2026 Jun 26;17(37):10702–7. doi: 10.1021/acs.jpclett.6c01422 (PMC13592068; doi:10.1021/acs.jpclett.6c01422)
Supplement: Supplementary file 1 [file jz6c01422_si_001.pdf]

Supporting Information

Detecting Vibrational Energy Transfer into an Enzyme Active Site via a Transition State Analog

*Jan G. Löffler, Erhan Deniz, Yunxia Shen, Carolin Feid, and Jens Bredenbeck**

*Institute of Biophysics, Johann Wolfgang Goethe-University, Max-von-Laue-Straße 1, 60438 Frankfurt am Main, Germany; Email: bredenbeck@biophysik.org

Contents:

I. Derivation of $\Delta x'_{min}$

II. Supplementary figures
Figure S1: Positions of AzAla and N_3^- in the FDH dimer.
Figure S2: CD spectra of wild-type FDH and FDH-Y341AzAla.
Figure S3: Background corrected FTIR spectra of N_3^- and the protein samples with Lorentzian fits.
Figure S4: TRIR background correction.
Figure S5: The VET signal's extrema and their separation.

III. Supplementary tables
Table S1: Parameters determined from the fits of the FTIR spectra.
Table S2: Comparison of spectral characteristics of N_3^- and other VET probes.

I. Derivation of $\Delta x'_{min}$

Given a Lorentzian function $f(x)$ centered around 0 with area a, and FWHM w:

$$f(x) = \frac{2a}{\pi}\frac{w}{4x^2 + w^2} = c\frac{w}{4x^2 + w^2} \tag{1}$$

The difference signal $d(x)$ generated by subtracting this Lorentzian from a Lorentzian shifted by -x_s is:

$$d(x) = c\left(\frac{w}{4(x + x_s)^2 + w^2} - \frac{w}{4x^2 + w^2}\right) \tag{2}$$

The x values of the extrema x' are the roots of the first derivative $d'(x)$:

$$d'(x) = c\left(\frac{8wx}{(4x^2 + w^2)^2} - \frac{8w(x + x_s)}{(4(x + x_s)^2 + w^2)^2}\right) \tag{3}$$

$$x'_1 = \frac{\sqrt{\frac{2\sqrt{x_s^4 + w^2x_s^2 + w^4}}{3} + \frac{x_s^2 - w^2}{3}}}{2} - \frac{x_s}{2} \tag{4}$$

$$x'_2 = -\frac{\sqrt{\frac{2\sqrt{x_s^4 + w^2x_s^2 + w^4}}{3} + \frac{x_s^2 - w^2}{3}}}{2} - \frac{x_s}{2} \tag{5}$$

$$x'_3 = \frac{\sqrt{-\frac{2\sqrt{x_s^4 + w^2x_s^2 + w^4}}{3} + \frac{x_s^2 - w^2}{3}}}{2} - \frac{x_s}{2} \tag{6}$$

$$x'_4 = -\frac{\sqrt{-\frac{2\sqrt{x_s^4 + w^2x_s^2 + w^4}}{3} + \frac{x_s^2 - w^2}{3}}}{2} - \frac{x_s}{2} \tag{7}$$

The separation $\Delta x'$ of the real-valued coordinates x'_1 and x'_2 in the limit of small shifts is:

$$\begin{aligned}\Delta x'_{min} &= \lim_{x_s \to 0}\sqrt{\frac{2\sqrt{x_s^4 + w^2x_s^2 + w^4}}{3} + \frac{x_s^2 - w^2}{3}} \\ &= \frac{w}{\sqrt{3}}\end{aligned} \tag{8}$$

Note: this is also the separation of the inflection points of $f(x)$ at $\pm\frac{w}{2\sqrt{3}}$, because:

$$f'(x) = \lim_{x_s \to 0}\frac{f(x + x_s) - f(x)}{x_s} = \lim_{x_s \to 0}\frac{d(x)}{x_s} \tag{9}$$

The result for the difference signal of a Gaussian function is:

$$\Delta x'_{min} = \frac{w}{\sqrt{\ln 4}} \tag{10}$$

II. Supplementary figures

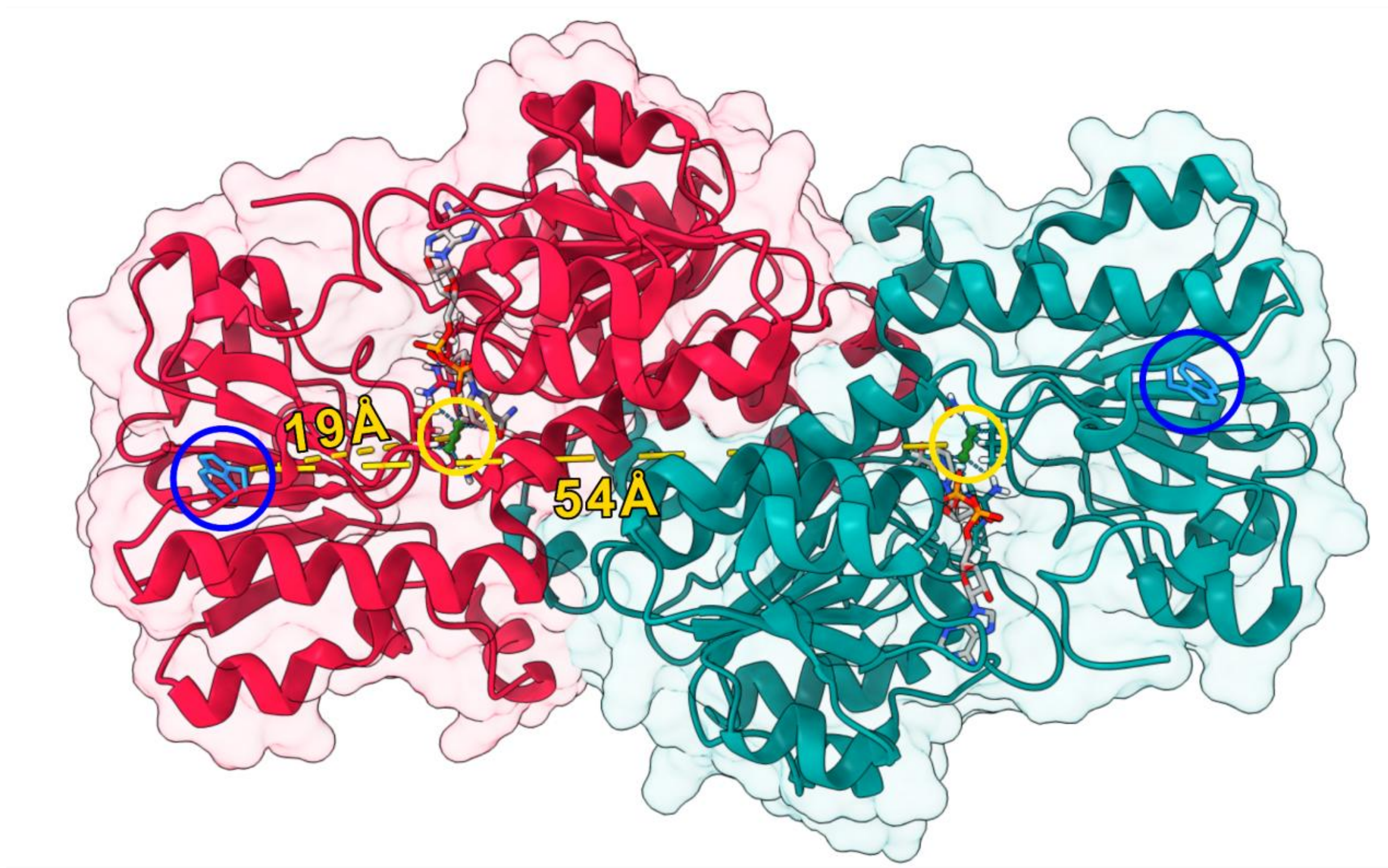

Figure S1. View of the FDH-AzAla341 (blue circles) dimer with, N_3^- (yellow circles), and NAD^+; illustrating the intra and inter subunit distances (yellow dashed line) between VET donor and VET sensor. The image is based on PDB: 5DN9[2] and was created in ChimeraX[3].

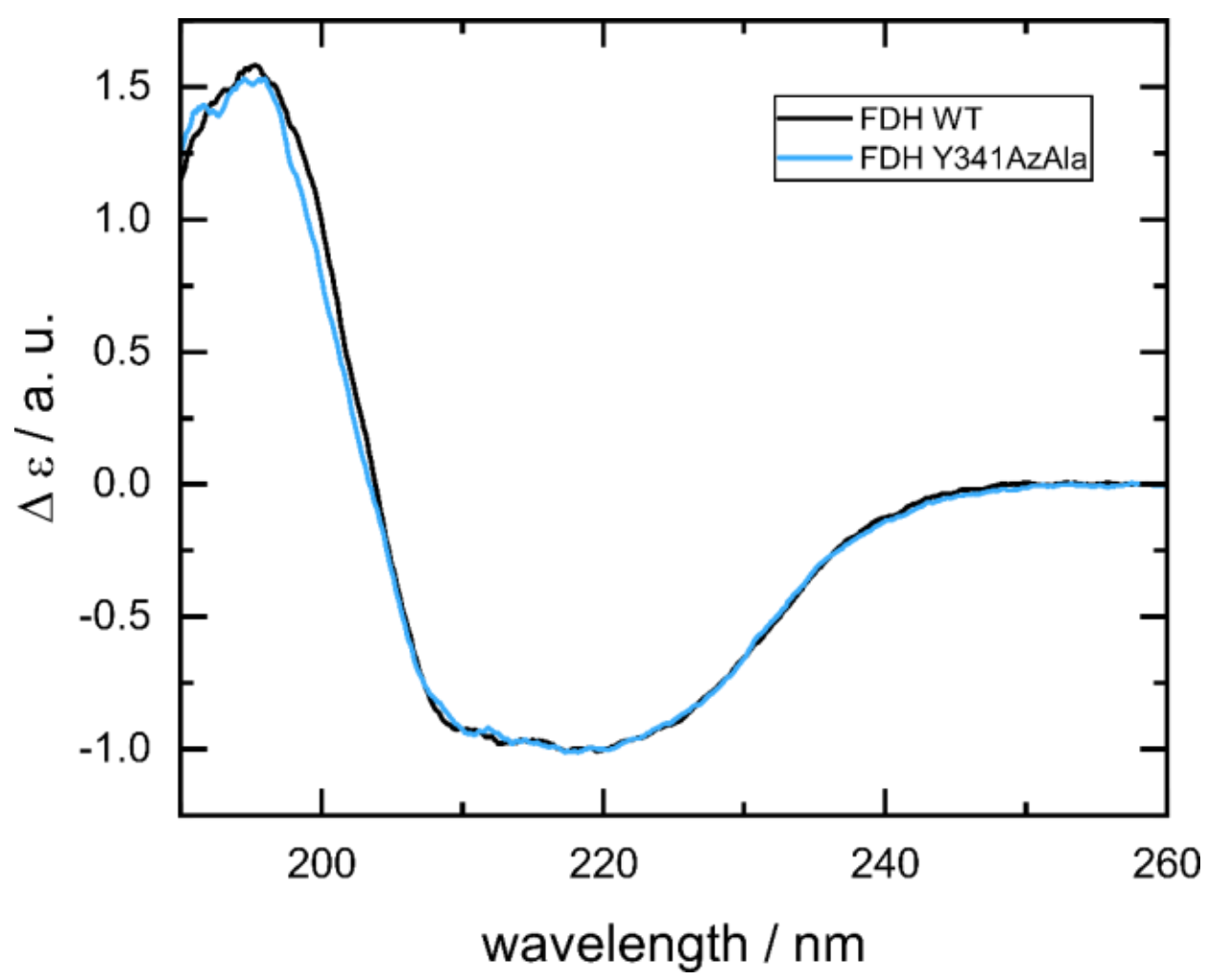

Figure S2. CD spectra of wild-type FDH and the FDH-Y341AzAla mutant, normalized to the signal at 220 nm. The close similarity of the spectra indicates that the secondary structure of the protein is not altered by the AzAla insertion.

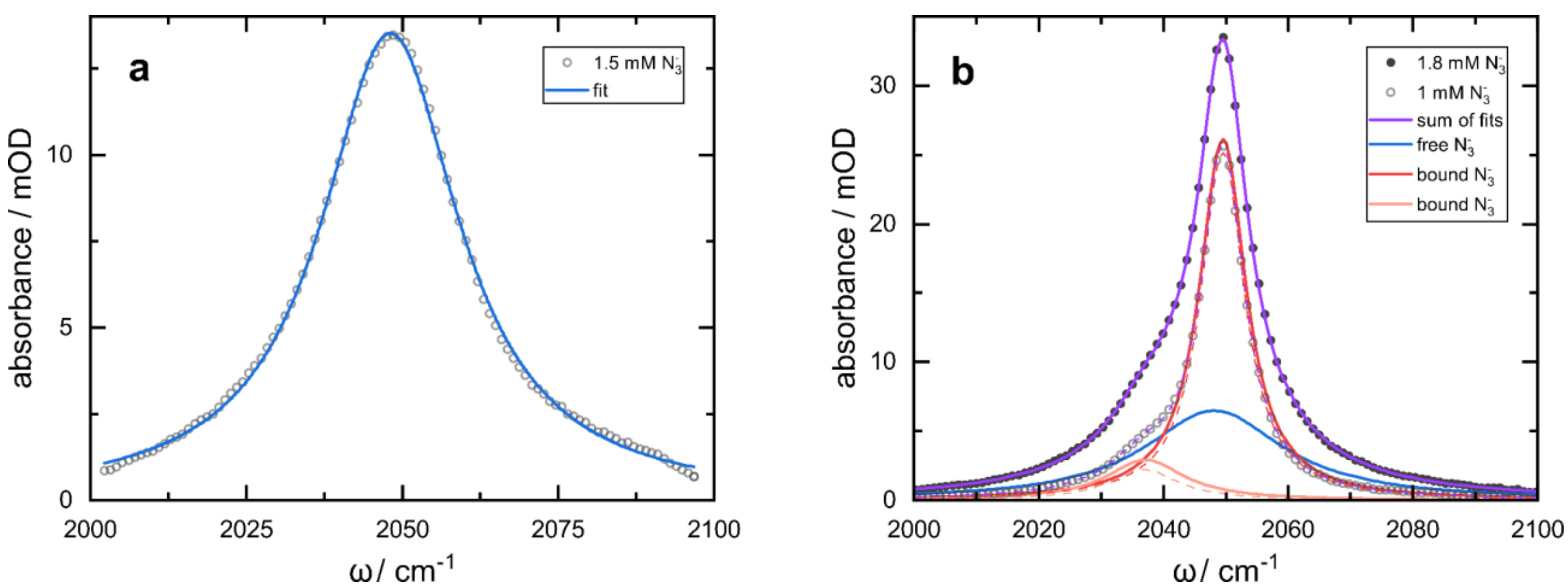

Figure S3. (a) Background corrected FTIR spectrum of NaN_3 in buffer and a Lorentzian fit to the data. (b) Background corrected FTIR spectra of the FDH-Y341AzAla laser sample with 1 mM (open circles) and 1.8 mM (filled circles) N_3^- and fits to these data as dashed and solid lines, respectively. Addition of more than 1 mM N_3^- mainly increases the component of the free N_3^-, indicating saturation of the protein.

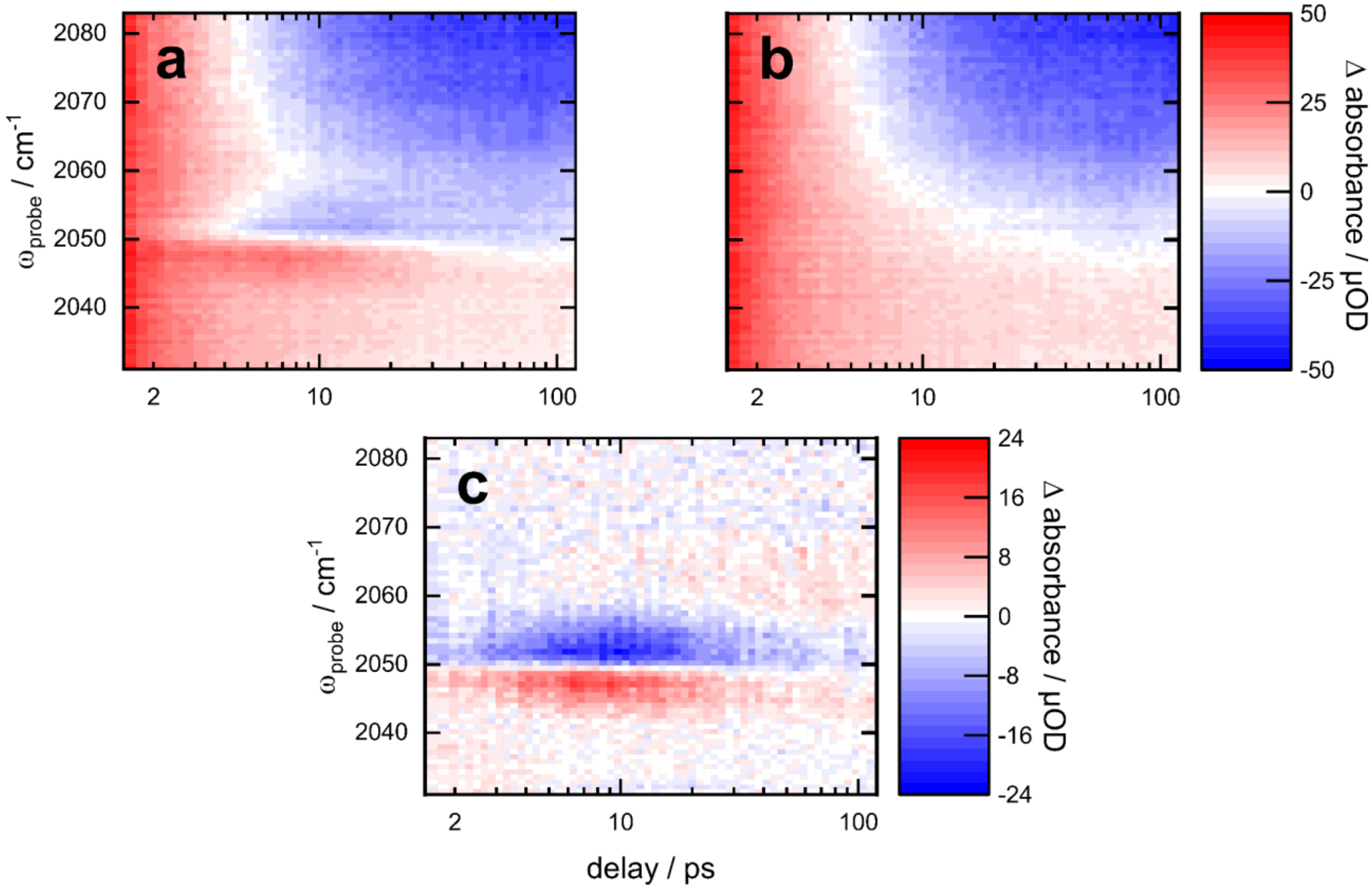

Figure S4. Background correction of the TRIR data. (a) TRIR data of the FDH-Y341AzAla in complex with NAD^+ and N_3^-. (b) TRIR data of FDH-Y341AzAla with NAD^+ but no N_3^-. The broad signal stems from energy transfer to the water. (c) Result of subtracting (b) from (a) with scaling and an offset parameter for each delay, excluding the signal pixels from the fit.[1]

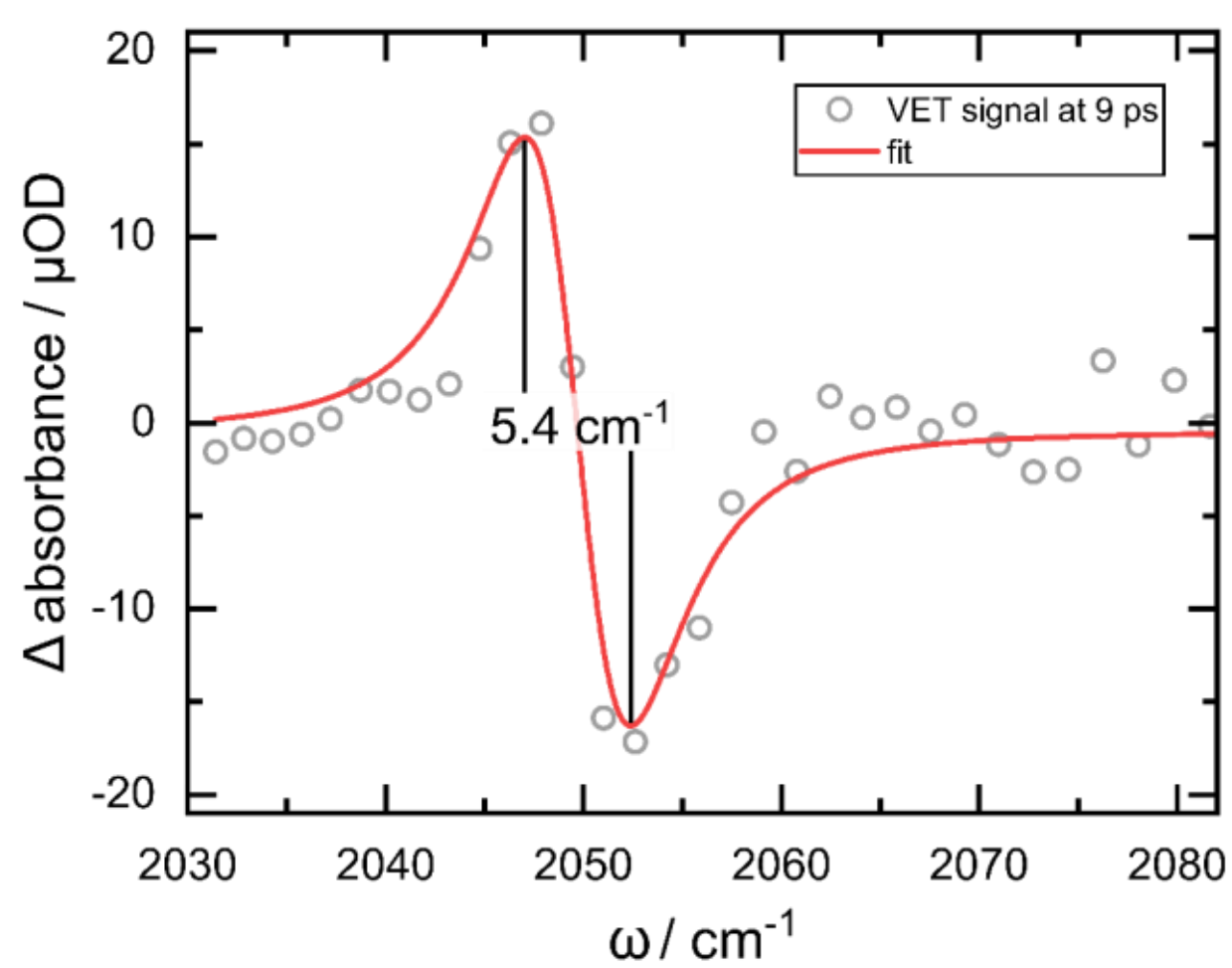

Figure S5. VET signal of N_3^- in FDH-Y341AzAla at 9 ps delay. A fit of the difference of two Lorentzian functions (FWHM = 9 cm^{-1}) to the signal results in a separation of the peaks of $\Delta x' = 5.4$ cm^{-1}.

III. Supplementary tables

Table S1. Fitting parameters (and their standard errors) of Lorentzian fits to the N_3^- absorption bands. The ω_c and FWHM of the free N_3^- absorption bands in the protein samples have been fixed to the values determined from N_3^- in buffer.

	N_3^- in buffer	FDH-Y341AzAla 1 mM N_3^-		FDH-Y341AzAla 1.8 mM N_3^-	
	free N_3^-	bound N_3^-	bound N_3^-	bound N_3^-	bound N_3^-
ω_c [cm^{-1}]	2048.10 ± 0.03	2049.496 ± 0.007	2036.77 ± 0.11	2049.514 ± 0.007	2037.11 ± 0.11
FWHM [cm^{-1}]	26.97 ± 0.14	8.48 ± 0.05	13.5 ± 0.4	9.02 ± 0.05	15.3 ± 0.4
Amplitude [mOD]		25.2 ± 0.2	2.24 ± 0.06	26.14 ± 0.13	2.92 ± 0.06

Table S2. Comparison of spectral characteristics of N_3^- and other VET probes in aqueous solution.

	extinction coefficient [M^{-1} cm^{-1}]	FWHM [cm^{-1}]	common occurrence of Fermi resonances
N_3^-	1600	27	No
Azidoalanine[4]	417	28.6	Yes
Azidohomoalanine[4]	405	31.4	Yes
Azidophenylalanine[4]	388	34.5	Yes
Cyanophenylalanine[4]	224	10.2	No
Cyanotryptophan[4]	109	12.2	No

REFERENCES

1. E. Deniz, J. G. Löffler, A. Kondratiev, A. R. Thun, Y. Shen, G. Wille, and J. Bredenbeck, Rev. Sci. Instrum. **93,** 33001 (2022).
2. Q. Guo, L. Gakhar, K. Wickersham, K. Francis, A. Vardi-Kilshtain, D. T. Major, C. M. Cheatum, and A. Kohen, Biochemistry **55,** 2760 (2016).
3. T. D. Goddard, C. C. Huang, E. C. Meng, E. F. Pettersen, G. S. Couch, J. H. Morris, and T. E. Ferrin, Protein Sci. **27,** 14 (2018).
4. J. G. Löffler, E. Deniz, C. Feid, V. G. Franz, and J. Bredenbeck, Angew. Chem. Int. Ed. **61**, e202200648 (2022).
